# Cause-specific mortality of children younger than 5 years in communities receiving biannual mass azithromycin treatment in Niger: verbal autopsy results from a cluster-randomised controlled trial

**DOI:** 10.1016/S2214-109X(19)30540-6

**Published:** 2020-01-22

**Authors:** Jeremy D Keenan, Ahmed M Arzika, Ramatou Maliki, Sanoussi Elh Adamou, Fatima Ibrahim, Mariama Kiemago, Nana Fatima Galo, Elodie Lebas, Catherine Cook, Benjamin Vanderschelden, Robin L Bailey, Sheila K West, Travis C Porco, Thomas M Lietman, MORDOR-Niger Study Group, Paul M Emerson, Jerusha Weaver, Sheila K West, Robin L Bailey, John Hart, Amza Abdou, Boubacar Kadri, Nassirou Beido, E Kelly Callahan, Aisha E Stewart, Ahmed M Arzika, Sanoussi Elh Adamou, Nana Fatima Galo, Fatima Ibrahim, Salissou Kane, Mariama Kiemago, Ramatou Maliki, Catherine Cook, Sun Y Cotter, Thuy Doan, Dionna M Fry, Jeremy D Keenan, Elodie Lebas, Thomas M Lietman, Ying Lin, Kieran S O'Brien, Catherine E Oldenburg, Travis C Porco, Kathryn J Ray, Philip J Rosenthal, George W Rutherford, Benjamin Vanderschelden, Nicole E Varnado, Lina Zhong, Zhaoxia Zhou

**Affiliations:** a Francis I Proctor Foundation, University of California, San Francisco, CA, USA; b Department of Ophthalmology, University of California, San Francisco, CA, USA; c Department of Epidemiology & Biostatistics, University of California, San Francisco, CA, USA; d Institute for Global Health Sciences, University of California, San Francisco, CA, USA; e The Carter Center Niger, Niamey, Niger; f London School of Hygiene & Tropical Medicine, London, UK; g Dana Center for Preventive Ophthalmology, Wilmer Eye Institute, Johns Hopkins University, Baltimore, MD, USA

## Abstract

**Background:**

The Macrolides Oraux pour Réduire les Décès avec un Oeil sur la Résistance (MORDOR) trial found that biannual mass distribution of azithromycin to children younger than 5 years in Niger reduced the primary outcome of all-cause mortality by 18%. We aimed to determine the causes of mortality among deceased children using verbal autopsy.

**Methods:**

In this 2-year cluster-randomised controlled trial, 594 community clusters in Niger were randomly allocated (1:1 ratio) to receive biannual mass distributions of either oral azithromycin (approximately 20 mg per kg of bodyweight) or placebo targeted to children aged 1–59 months. Participants, study investigators, and field workers were masked to treatment allocation. Between Nov 23, 2014, and July 31, 2017, 3615 child deaths were recorded by use of biannual house-to-house censuses, and verbal autopsies were done between May 26, 2015, and May 17, 2018, to identify cause of death. Cause-specific mortality, as assessed by verbal autopsy, was a prespecified secondary outcome. This trial is completed and is registered with ClinicalTrials.gov, NCT02047981.

**Findings:**

Between Nov 23, 2014, and July 31, 2017, 303 communities (n=40 375 children at baseline) in Niger received mass azithromycin and 291 communities (n=35 747 children at baseline) received placebo. Treatment coverage was 90·3% (SD 10·6) in the azithromycin group and 90·4% (10·1) in the placebo group. No communities were lost to follow-up. In total, 1727 child deaths in the azithromycin group and 1888 child deaths in the placebo group were reported from the population censuses. Of these, the cause of death for 1566 (90·7%) children in the azithromycin group and 1735 (91·9%) children in the placebo group were ascertained by verbal autopsy interviews. In the azithromycin group, 437 (27·9%) deaths were due to malaria, 252 (16·1%) deaths were due to pneumonia, and 234 (14·9%) deaths were due to diarrhoea. In the placebo group, 493 (28·4%) deaths were due to malaria, 275 (15·9%) deaths were due to pneumonia, and 251 (14·5%) deaths were due to diarrhoea. Relative to communities that received placebo, child mortality in communities that received azithromycin was lower for malaria (incidence rate ratio 0·78, 95% CI 0·66–0·92; p=0·0029), dysentery (0·65, 0·44–0·94; p=0·025), meningitis (0·67, 0·46–0·97; p=0·036), and pneumonia (0·83, 0·68–1·00; p=0·051). The distribution of causes of death did not differ significantly between the two study groups (p=0·98).

**Interpretation:**

Mass azithromycin distribution resulted in approximately a third fewer deaths in children aged 1–59 months due to meningitis and dysentery, and a fifth fewer deaths due to malaria and pneumonia. The lack of difference in the distribution of causes of death between the azithromycin and placebo groups could be attributable to the broad spectrum of azithromycin activity and the study setting, in which most childhood deaths were due to infections.

**Funding:**

Bill & Melinda Gates Foundation.

## Introduction

Despite considerable improvements over the past few decades, childhood mortality remains far above the Sustainable Development Goals for many regions in sub-Saharan Africa.^1^, ^2^ Most childhood deaths in Africa are due to infectious diseases.^2^ The increasing availability of vaccines, coupled with improvements in primary health-care systems, promises to further increase childhood survival.^3^, ^4^ Nevertheless, additional interventions to further reduce childhood mortality would be welcome.

The Macrolides Oraux pour Réduire les Décès avec un Oeil sur la Résistance (MORDOR) cluster-randomised trial was done in three study sites located in sub-Saharan Africa: Malawi, Niger, and Tanzania. Children aged 1–59 months in communities in these three countries were randomly assigned to receive biannual mass treatment of either azithromycin or placebo to determine the efficacy of mass antibiotic distributions for reducing childhood mortality. Cluster randomisation was chosen because antibiotic consumption could have direct effects on treated individuals and also indirect spillover effects on untreated children (ie, herd protection).^5^ All-cause mortality among children younger than 5 years was approximately 14% lower in communities that received azithromycin than in those that received placebo across all sites.^6^ The largest effect was observed in Niger, where all-cause mortality was 18% lower in communities that received azithromycin than in those that received placebo. However, cause-specific mortality was not explored in depth.^6^ In this study, we aimed to compare cause-specific mortality in children younger than 5 years in Niger between the two study groups using verbal autopsy results. Cause-specific mortality was a prespecified secondary outcome of the MORDOR trial. We hypothesised that child deaths due to infectious diseases would be less common in communities that received azithromycin than in communities that received placebo.

**Research in context**

**Evidence before this study**

The Macrolides Oraux pour Réduire les Décès avec un Oeil sur la Résistance (MORDOR) trial was a cluster-randomised trial of mass biannual azithromycin administration in three countries in sub-Saharan Africa. The results showed a 13·5% overall reduction in mortality in children aged 1–59 months in communities that received azithromycin twice yearly.

**Added value of this study**

We report verbal autopsy results for childhood deaths that occurred in communities located at the Niger site of the MORDOR trial. Fewer childhood deaths due to malaria, dysentery, meningitis, and pneumonia were reported in communities that received azithromycin than in communities that received placebo; however, no difference in the overall distribution of causes of death was found between the two study groups.

**Implications of all the available evidence**

Community-wide biannual azithromycin distribution reduced the number of childhood deaths attributable to infectious diseases in Niger. The lack of difference in the distribution of causes of death between communities that received azithromycin and those that received placebo could be attributable to the broad spectrum of azithromycin activity.

## Methods

### Study design and participants

The MORDOR trial was a parallel-group, cluster-randomised trial in which communities in Malawi, Niger, and Tanzania were randomly assigned to receive biannual mass distributions of either oral azithromycin or placebo between Nov 23, 2014, and July 31, 2017, with study drugs distributed to all children aged 1–59 months in the community. Children were monitored approximately every 6 months by use of a population census. A shortened verbal autopsy was done at each country's study site for child deaths reported in the census by use of a standardised questionnaire, the results for which have been reported separately.^6^ Although the shortened verbal autopsy was the only cause of death assessment required across the study sites of all three countries, individual study sites were allowed to do more intensive assessments if desired. Thus, in addition to the shortened verbal autopsy questionnaire, the full 2007 WHO verbal autopsy questionnaire was also done in Niger to provide a more detailed account of the cause of death of children in each study group. In this study, we report the causes of child deaths in Niger, as assessed from this full questionnaire. No changes were made to the study design or sample size after the trial commenced.

The unit of randomisation in Niger was the grappe: a government-defined demographic unit analogous to a village. Communities located in the Boboye or Loga departments with a population of 200–2000 citizens, according to the most recent (2012) government census, were eligible for enrolment. A study census was done in each community approximately every 6 months commencing from Nov 23, 2014, up to July 31, 2017. Children aged 1–59 months identified in the census were eligible for treatment with azithromycin or placebo, and their vital status was updated at the following census. Children weighing less than 3800 g were excluded from treatment, but not from the census. If an eligible child was reported as having died, a verbal autopsy was attempted. Verbal autopsies were done between May 26, 2015, and May 17, 2018. It should be noted that, although this study activity was termed a census because of the door-to-door nature of data collection, this activity was analogous to a cohort with open enrolment and repeated follow-up visits. Ethical approval was obtained from the Committee on Human Research at the University of California, San Francisco, and the Institutional Review Board of the Nigerien Ministry of Health. Caregivers of children provided oral informed consent for treatments and verbal autopsies.

### Randomisation and masking

Community clusters in Niger were randomly assigned (1:1) to either the azithromycin group or the placebo group. The trial biostatistician generated the random allocation sequence without restriction using the R statistical package (version 3). The Nigerien study coordinator enrolled communities and assigned them to receive the allocated intervention, and also coordinated all aspects of the trial before and after randomisation. The trial biostatistician analysed the results of the trial. Allocation concealment (ie, preventing study investigators responsible for enrolling participants from knowing which participant would receive which treatment) was done at the cluster level by enrolling all communities before randomisation, and concealment was done at the individual level by administering the treatment to all eligible children. Bottles were labelled with one of ten treatment letters, with five letters corresponding to azithromycin and five letters corresponding to placebo. Study bottles, packaging, and the appearance of the drug were identical between the two treatment groups. Apart from the trial biostatistician, all study participants, field personnel, and investigators were masked to treatment allocation.

### Procedures

Study drugs consisted of either azithromycin powder for suspension (200 mg/5 mL) or an identically packaged placebo powder (both provided by Pfizer). Children aged 1–59 months were given a volume of suspension corresponding to at least 20 mg/kg azithromycin or the analogous volume of placebo. Suspension volumes were estimated by use of a height stick for children who could stand and were estimated by weight with a digital hanging scale (American Weigh Scales, Cumming, GA, USA) for children who could not stand.^7^ Drug administration was directly observed by study personnel. Caregivers (ie, the adult responsible for the child, usually a parent) were told to contact a community representative if adverse events occurred within 7 days of the child receiving the drug. A formal adverse events survey was done for study participants aged 1–5 months.^8^

The 2007 WHO questionnaire for children aged from 4 weeks to 14 years was used for the verbal autopsy. This instrument was chosen because it was the most widely used survey at the time the study was started (2014).^9^ The primary caregiver was interviewed at their household by medical officers masked to the treatment groups. All interviewers received biannual training. Seasonal migration is common in the study area of Niger (Loga and Boboye); therefore locating the households of deceased children was sometimes difficult. Before a child was considered absent or missing, at least three separate visits had to be made to their household. The date of death was ascertained independently from both the verbal autopsy and the census (with an intraclass correlation coefficient of 0·88 [95% CI 0·87–0·89] comparing date estimates from the two collection methods).

Cause of death was ascertained from the verbal autopsy interviews in three ways. First, a single cause of death was assigned to each child by use of an adapted version of a pre-defined algorithm described previously (appendix pp 3–5).^10^, ^11^ The algorithm had been tested in a separate study^10^ in Niger and was found to provide a distribution of cause of death similar to that of physician coding. This algorithm first classified the presence or absence of one of 17 potential causes of death, and then it assigned the primary cause of death on the basis of a hierarchy. Four causes of death (diarrhoea, dysentery, malaria, and pneumonia) were subclassified as possible or probable by the algorithm. Possible and probable deaths were ultimately combined when classifying the final cause of death for statistical analyses. Similarly, malnutrition was subclassified into two types: underlying malnutrition, whereby the clinical signs occurred as the first element of the illness, and non-underlying, whereby the clinical signs occurred later. These subclassifications of malnutrition were ultimately combined when classifying the final cause of death. Second, the algorithm was implemented without the hierarchy, allowing multiple causes of death to be assigned to a child. Finally, the raw verbal autopsy interview responses were treated as a multivariate outcome using the answers to all dichotomous questions that were required from all respondents (appendix p 6).

### Outcomes

The primary outcome of the MORDOR trial was all-cause mortality among children aged 1–59 months, as assessed biannually by use of a door-to-door census. The results of the primary outcome have been published previously.^6^ A prespecified secondary outcome was cause of death as assessed by verbal autopsy for all deaths identified during the census. Verbal autopsies also functioned as validation of vital status reporting from the census. However, the prespecified primary outcome of the trial was based only on the information obtained from the census and was not updated with any additional information obtained from the verbal autopsy.

### Statistical analysis

The four intercensal periods of the study, which took place from Nov 23, 2014, to July 31, 2017, were analysed separately. Deaths were counted only if the child had been documented as alive and living in the household at the biannual census 6 months previously.^6^ The distribution of causes of death between the two study groups were compared by use of a permuted χ^2^ test with Monte Carlo resampling (10 000 permutations at the community level; prespecified primary analysis). In a prespecified secondary multivariate analysis, the hierarchy was not applied and each child was instead allowed to be assigned multiple causes of death (ie, a binary response for each of the 12 individual causes of death plus a binary indicator for an unspecified cause), with a permutational MANOVA (PERMANOVA) used to analyse differences between the two study groups (Euclidean distance matrix, 10 000 permutations at the community level). We chose to use a PERMANOVA because this statistical technique accommodates multiple response variables using a non-parametric permutation approach.^12^ A similar PERMANOVA test was used to analyse the raw verbal autopsy responses. In additional secondary analyses, the incidence rate ratio (IRR) for each cause of death was calculated from a negative binomial regression that modelled the number of cause-specific deaths per community as a function of the randomisation group, with total person-time as an offset. Negative binomial regression was chosen to model the count data because this method contains a parameter to adjust for overdispersion (eg, an excessive number of zeros).^13^ R version 3 was used for statistical analyses. Sample size calculations for the trial were based on the primary all-cause mortality outcome.^6^ Inclusion of 290 communities in each study group would provide 80% power to detect a 30% relative difference in any specific cause of death between the two study groups, assuming a mortality rate of 0·5% per year (ie, five deaths per 1000 person-years) in the placebo group, a coefficient of variation of 0·51, a population of 111 children per community, 10% loss to follow up, and an α level of 0·05. A data safety and monitoring committee oversaw the study (appendix p 2). This study is registered with ClinicalTrials.gov, NCT02047981.

### Role of the funding source

The funder of the study had no role in study design, data collection, data analysis, data interpretation, or writing of the report. The corresponding author had full access to all the data in the study and had final responsibility for the decision to submit for publication.

## Results

**Figure 1:**
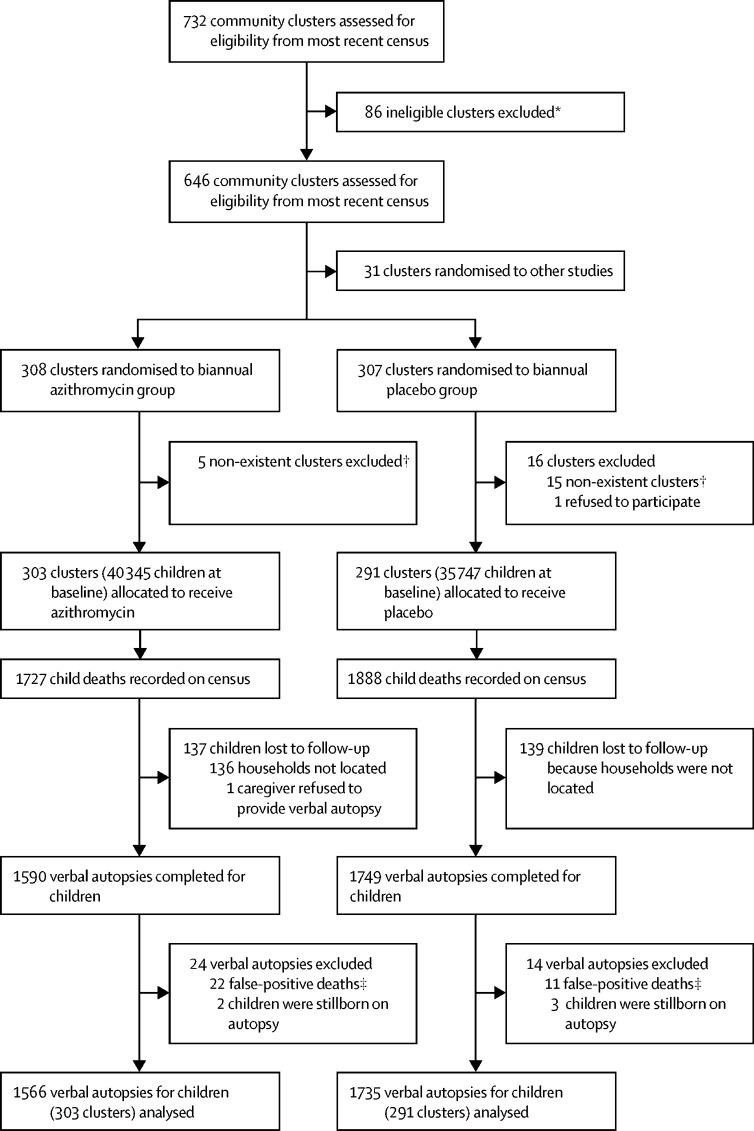
Trial profile The average number of children per cluster who received azithromycin or placebo and the average number of children per cluster who did not receive either treatment despite being randomised to that arm are provided in the appendix (p 7). *Ineligible clusters were those that had a population of fewer than 200 persons or more than 2000 persons on the most recent census, or communities present on the most recent government census but not required for the target sample size of the study. †Refers to communities on the government census that no longer existed by the time the study census was done. ‡False-positive deaths included children who were recorded as deceased on the census but were recorded as alive on the verbal autopsy.

Between Nov 23, 2014, and July 31, 2017, 303 communities in the Niger site of the MORDOR trial received biannual mass azithromycin distributions and 291 communities received biannual mass placebo distributions targeted to children aged 1–59 months (figure 1). At baseline, 40 345 children were enrolled in the azithromycin group and 35 747 children in the placebo group (table 1). Treatment coverage was high in both groups, with a mean coverage of 90·3% (SD 10·6) in communities assigned to the azithromycin group and 90·4% (10·1) in communities assigned to the placebo group across the four mass drug distribution periods (appendix p 7). No hospitalisations or life-threatening illnesses were reported as a result of study medication in either group over the duration of the study, although most children had no access to a hospital.

**Table 1:** Baseline characteristics of communities from the initial census

		**Azithromycin group (303 communities)**	**Placebo group (291 communities)**
Mean number of children per community		133 (93)	123 (88)
Percentage of children by age group			
	1–11 months	20·2% (6·1)	20·5% (6·1)
	12–23 months	17·0% (5·1)	16·8% (5·3)
	24–35 months	21·2% (4·9)	21·0% (5·2)
	36–47 months	21·7% (5·6)	21·1% (4·8)
	48–59 months	19·9% (10·5)	20·6% (10·2)
Percentage of male children		51·2% (6·2)	51·4% (6·4)

Data are mean (SD).

Over 2 years of biannual censuses, 1727 child deaths were documented in the azithromycin group, and 1888 child deaths were documented in the placebo group. Verbal autopsy interviews were completed for 1590 (92·1%) child deaths in the azithromycin group and 1749 (92·6%) child deaths in the placebo group. The median time from the reported date of death until completion of the verbal autopsy was 6 months (IQR 2–13) in the azithromycin group and 7 months (3–14) in the placebo group. The verbal autopsy confirmed the census death outcome (ie, a child reported as alive at an initial census and then deceased at the subsequent census) for 3301 (98·9%) of 3339 cases, with the remainder of verbal autopsies reporting a stillbirth or miscarriage (n=5 [0·1%]), or a child who had not died (n=33 [1·0%]; table 2). Discrepancies between the census and verbal autopsy for the reported vital status of children were slightly more common in the azithromycin group (22 [1·3%] of 1727 children identified as having died in the census were found to be alive during the verbal autopsy) than the placebo group (11 [0·6%] of 1888 children identified as having died in the census were found to be alive during the verbal autopsy).

**Table 2:** Outcomes of the verbal autopsy interview

	**Azithromycin group (n=1727)**	**Placebo group (n=1888)**
**Verbal autopsy not performed**		
Household not located	136 (7·9%)	139 (7·4%)
Caregiver refused	1 (0·1%)	0 (0%)
**Verbal autopsy performed**		
Child reported as alive	22 (1·3%)	11 (0·6%)
Child reported as stillborn or miscarriage	2 (0·1%)	3 (0·2%)
Cause of death ascertained	1566 (90·7%)	1735 (91·9%)

Data are n (%).

**Figure 2:**
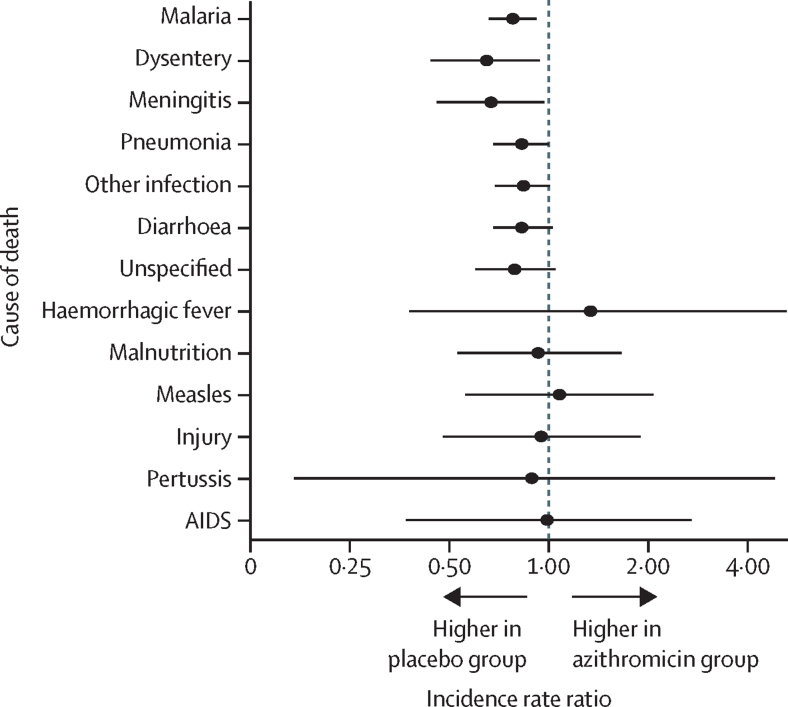
Forest plot showing the effect of mass azithromycin distribution on cause-specific mortality in children younger than 5 years in Niger The incidence rate ratio estimates and 95% CIs are shown for each cause of death, as determined from the verbal autopsy. The estimates are ordered by degree of statistical significance (from strongest association to weakest association).

Table 3 shows the primary cause of death in children (as identified by the pre-defined algorithm) and the incidence estimates for each cause of death. Figure 2 shows the effect of azithromycin treatment on cause-specific mortality in children relative to placebo, with causes of death listed in descending order of strength of association. Relative to mass placebo distribution, mass azithromycin distribution was most significantly associated with a reduction in the number of child deaths due to malaria (IRR 0·78, 95% CI 0·66–0·92; p=0·0029), followed by dysentery (0·65, 0·44–0·94; p=0·025), meningitis (0·67, 0·46–0·97; p=0·036), and pneumonia (0·83, 0·68–1·00; p=0·051). Mass azithromycin distribution had no significant effect on the number of child deaths due to viruses (ie, AIDS, haemorrhagic fever, and measles) or non-infectious causes (ie, injury and malnutrition; figure 2), and when these five causes were pooled together, cause-specific mortality was similar between the two treatment groups (1·00, 0·72–1·40; p=0·981).

**Table 3:** Primary cause of death ascertained from verbal autopsy

		**Azithromycin group**		**Placebo group**		**Incidence rate ratio (95% CI)**	**p value**
		Number of children (%)	Incidence (95% CI)	Number of children (%)	Incidence (95% CI)		
**Non-infectious cause**							
Injury		17 (1·1%)	0·22 (0·14–0·36)	16 (0·9%)	0·23 (0·14–0·38)	0·95 (0·48–1·90)	0·883
Malnutrition*		31 (2·0%)	0·39 (0·26–0·59)	29 (1·7%)	0·42 (0·28–0·63)	0·93 (0·53–1·66)	0·816
	Underlying	4 (0·3%)	0·05 (0·02–0·14)	6 (0·3%)	0·09 (0·04–0·19)	0·60 (0·15–2·09)	0·423
	Non-underlying	27 (1·7%)	0·35 (0·23–0·53)	23 (1·3%)	0·33 (0·21–0·52)	1·05 (0·57–1·95)	0·881
**Infectious cause**							
AIDS		9 (0·6%)	0·12 (0·06–0·23)	8 (0·5%)	0·12 (0·06–0·24)	0·99 (0·37–2·71)	0·990
Measles		23 (1·5%)	0·30 (0·19–0·46)	19 (1·1%)	0·28 (0·17–0·45)	1·08 (0·56–2·08)	0·823
Meningitis		63 (4·0%)	0·82 (0·63–1·09)	83 (4·8%)	1·22 (0·96–1·57)	0·67 (0·46–0·97)	0·036
Dysentery		57 (3·6%)	0·74 (0·55–0·98)	77 (4·4%)	1·14 (0·88–1·47)	0·65 (0·44–0·94)	0·025
	Probable	21 (1·3%)	0·28 (0·18–0·43)	38 (2·2%)	0·56 (0·40–0·79)	0·49 (0·27–0·86)	0·014
	Possible	36 (2·3%)	0·47 (0·33–0·65)	39 (2·2%)	0·57 (0·41–0·78)	0·82 (0·51–1·30)	0·399
Diarrhoea		234 (14·9%)	3·01 (2·59–3·49)	251 (14·5%)	3·60 (3·11–4·17)	0·83 (0·68–1·03)	0·088
	Probable	104 (6·6%)	1·37 (1·10–1·70)	114 (6·6%)	1·66 (1·34–2·05)	0·82 (0·61–1·12)	0·214
	Possible	130 (8·3%)	1·65 (1·36–2·01)	137 (7·9%)	1·96 (1·61–2·36)	0·84 (0·65–1·11)	0·223
Pertussis		3 (0·2%)	0·04 (0·01–0·12)	3 (0·2%)	0·04 (0·01–0·14)	0·89 (0·17–4·83)	0·891
Pneumonia		252 (16·1%)	3·27 (2·85–3·75)	275 (15·9%)	3·96 (3·46–4·53)	0·83 (0·68–1·00)	0·051
	Probable	81 (5·2%)	1·06 (0·83–1·36)	97 (5·6%)	1·40 (1·11–1·77)	0·76 (0·54–1·06)	0·107
	Possible	171 (10·9%)	2·22 (1·89–2·59)	178 (10·3%)	2·57 (2·20–3·01)	0·86 (0·69–1·07)	0·186
Malaria		437 (27·9%)	5·60 (4·97–6·32)	493 (28·4%)	7·22 (6·43–8·11)	0·78 (0·66–0·92)	0·003
	Probable	57 (3·6%)	0·74 (0·57–0·96)	53 (3·1%)	0·77 (0·59–1·01)	0·96 (0·66–1·40)	0·837
	Possible	380 (24·3%)	4·89 (4·29–5·57)	440 (25·4%)	6·45 (5·69–7·31)	0·76 (0·63–0·91)	0·003
Haemorrhagic fever		6 (0·4%)	0·08 (0·03–0·17)	4 (0·2%)	0·06 (0·02–0·16)	1·34 (0·38–5·25)	0·650
Other infection		325 (20·8%)	4·24 (3·70–4·85)	354 (20·4%)	5·06 (4·44–5·78)	0·84 (0·69–1·01)	0·063
Unspecified		109 (7·0%)	1·43 (1·17–1·75)	123 (7·1%)	1·81 (1·49–2·19)	0·79 (0·60–1·05)	0·104
Total		1566 (100·0%)	22·32 (20·86–23·87)	1735 (100%)	27·26 (25·50–29·13)	0·82 (0·75–0·90)	<0·001

Primary cause of death was assessed by use of a hierarchical algorithm. Incidence is reported as deaths per 1000 person-years, estimated from a negative binomial regression of community-level death counts, with total community person-time determined from the census used as the offset.

* Underlying malnutrition is defined by the occurrence of clinical signs of malnutrition as the first element of the illness, whereas clinical signs of malnutrition occur later in non-underlying malnutrition.

The distribution of child deaths did not differ between the two groups (p=0·980). This result remained not significant when child deaths attributable to probable or possible malnutrition were analysed separately (p=0·962), when infectious causes of death were compared with non-infectious causes (p=0·766), when multiple causes of death were allowed for each child (p=0·733), and when raw verbal autopsy responses were used (p=0·058).

## Discussion

The MORDOR trial was a placebo-controlled, community-randomised trial that found an 18% reduction in all-cause mortality among children aged 1–59 months in Nigerien communities that received biannual mass azithromycin treatment. The causes of death of children in this trial have been reported previously,^6^ but cause of death was ascertained in that report by use of a shortened verbal autopsy instrument. In this present study, we report the results from interviews done using the full 2007 WHO verbal autopsy instrument. The distribution of child deaths in the azithromycin and placebo groups in the present study was largely similar to the distribution of child deaths observed when the abbreviated verbal autopsy was used. However, the full verbal autopsy instrument detected fewer cases of malaria and more cases of respiratory infection than did the shortened instrument. Cause-specific mortality among children aged 1–59 months was significantly lower in the azithromycin group than the placebo group for several causes of death due to infectious diseases. However, the distribution of causes of death was not significantly different between the azithromycin and placebo group.

One reason verbal autopsies were conducted in the MORDOR trial was to validate child deaths reported in the censuses. An independent team of field personnel conducted the verbal autopsies, which were attempted for all deaths identified during the census. We found that a death identified during the census in Niger had a 98·8% positive predictive value based on the verbal autopsy results. False-positive deaths (ie, children who were reported as deceased on the census but were reported as alive on the verbal autopsy) were slightly more common in the azithromycin group, suggesting that biased ascertainment of child deaths during the census did not account for the observed reduction in child mortality in the azithromycin group versus the placebo group in the MORDOR trial.

We used the 2007 WHO verbal autopsy questionnaire combined with an expert hierarchical algorithm used in previous studies in Niger^9^, ^10^, ^11^ to assign a single cause of death to each child. Other instruments with a slightly different set of questions are available (eg, instruments from WHO, the Population Health Metrics Research Consortium, and the INDEPTH network, among others), as are alternate analysis methods, such as a physician review, different algorithms, and Bayesian methods.^14^, ^15^, ^16^, ^17^ None of the instruments or analysis techniques have perfect diagnostic accuracies, and each method is likely to misclassify a proportion of deaths, particularly those associated with vague or non-specific symptoms, such as malaria.^11^, ^18^, ^19^ Although it is possible that the use of a different questionnaire or analysis method would have resulted in slightly different estimates, the overall conclusions of our analysis are likely to be robust given the randomisation and masking methods used in the trial.

The distribution of the causes of death among children observed in the present study is consistent with other studies from Niger that have used verbal autopsy questionnaires. For example, Kalter and colleagues^10^ used a similar algorithmic approach to analyse 620 child deaths in Niger from 2007 to 2010 and found that the predominant causes of death among children aged 1–59 months were malaria (29%), diarrhoea (20%), meningitis (18%), and pneumonia (12%). By comparison, the predominant causes of death found in the present study were malaria (28%), diarrhoea (15%), pneumonia (16%), and meningitis (4%). Other studies^2^, ^20^ have reported similar estimates of the distribution of causes of childhood mortality in Niger, with mortality estimates for meningitis more consistent with those reported in our study than those from the study by Kalter and colleagues.^10^

We found that mass azithromycin distribution did not significantly alter the distribution of the causes of child deaths in Niger compared with mass distribution of placebo. One possible explanation for this result could be that most child deaths after the first month of life were due to infections, and the broad spectrum of activity of azithromycin acted against a range of bacterial and parasitic microorganisms responsible for these deaths, as suggested by our analysis. Thus, mass azithromycin distribution could have resulted in a small reduction in many causes of death due to infectious diseases, leading to a substantial reduction in the overall mortality of children, but only small changes in the proportion of deaths due to any specific cause.

The benefit of azithromycin was most pronounced for deaths due to malaria, meningitis, dysentery, and pneumonia. Although this finding could be explained, in part, because these diseases were the most common causes of death in children and therefore provided the most statistical power to detect a difference in treatment effect, azithromycin has a plausible treatment effect for each of these causes of death. Azithromycin has moderate anti-malarial activity because of its effects on the plasmodial apicoplast.^21^ Indeed, a sister trial done alongside the MORDOR trial found a reduction in the prevalence of malaria parasitaemia in communities that received azithromycin.^22^ The main causes of bacterial meningitis in Niger are *Neisseria meningitidis, Streptococcus pneumoniae*, and *Haemophilus influenzae*; *S pneumoniae* and *H influenzae* are also the major microorganisms that cause pneumonia.^23^, ^24^ The primary cause of bacillary dysentery is shigella, *Escherichia coli*, and *Campylobacter* spp.^25^ All of these bacteria are susceptible to azithromycin.

The mechanism of action by which mass azithromycin distribution reduces childhood mortality is unclear. Mass therapy with azithromycin has been speculated to clear established infections early in the disease course, thereby preventing mortality. Alternatively, azithromycin might clear commensal organisms (eg, nasopharyngeal pneumococcus), thus preventing the carriage of such organisms and the development of invasive infections. Azithromycin might also act as a prophylactic therapy that prevents children from acquiring an infection in the first instance. Besides these potential activities against specific pathogenic organisms, it is also possible that azithromycin serves a role as a modulator of the microbiota or of inflammatory mediators, which could, in turn, be protective against mortality. Alternatively, azithromycin could promote the growth of children by indirectly improving the health of their immune systems, leading to less severe infections. Ultimately, the mechanisms by which mass azithromycin distribution reduces mortality in children remains a matter of speculation, and further research is needed to identify the exact mechanisms.

Antibiotic resistance could theoretically reduce the activity of azithromycin for some bacterial pathogens, although macrolide antibiotics are not widely prescribed in Niger. Mass azithromycin distributions could select for resistant bacteria, although limiting the distribution of antibiotic treatments to children aged 1–59 months rather than to individuals of all ages (as is done in trachoma programmes) could reduce the antibiotic selection pressure.

Attributing a cause of death outside of the health-care system is difficult, especially in resource-limited settings.^26^ The most common technique currently used to address this problem is the verbal autopsy, whereby the caregivers of a deceased child are asked a series of questions designed to identify the signs of the final illness. However, this method of data collection has some shortcomings. Caregivers might not understand the terminology used in the verbal autopsy questionnaire, which could lead to incorrect reporting of the cause of death. For example, a previous study^27^ found that a fifth of neonatal deaths were misclassified as stillbirths—an error that also occurred in a few cases in the present study. In addition, caregivers might forget key details of the child's symptoms between the time of death and the interview. Furthermore, not all parents can be located after a child death is reported, which leads to incomplete information. Cause of death is established by the caregiver report, without any added information about vital signs or a physical examination by a health-care professional (eg, a medical doctor, medical officer, or a nurse). Many children will exhibit signs and symptoms of several different diseases; therefore, attributing a single cause of death can be difficult. Despite the limitations of the verbal autopsy, it is the most realistic option in resource-limited settings. Having cause of death determined by a health-care provider would be ideal, but was not feasible in the Niger study site, where few children are seen by a health-care provider during the final illness. Other methods, such as minimally invasive tissue sampling, could have provided more robust information; however, these tests were not included in the MORDOR trial, which was specifically designed to collect data on a small number of straightforward outcome measures from a large sample population.^28^, ^29^

Aside from the challenges of the verbal autopsy method, our study has several other limitations. As described above, the verbal autopsy method is subject to data misclassification during data collection and analysis. Although this error would limit statistical power, differential misclassification between treatment groups would not be expected because of the randomised nature of the study. A verbal autopsy interview was not completed for all child deaths, and it is possible that deaths lost to follow-up might have been systematically different from deaths for which a verbal autopsy was completed. However, the proportion of child deaths for which verbal autopsies were completed was approximately 90% in both groups, making differential loss to follow-up unlikely. The generalisability of the findings outside, and even within, Niger is unclear, especially for those infectious diseases with marked geospatial patterns, such as meningitis and malaria.

In summary, verbal autopsy results from the Niger site of the MORDOR trial revealed that mass azithromycin distribution to children aged 1–59 months had the most significant effect for deaths due to malaria, meningitis, dysentery, and pneumonia. Azithromycin has a plausible mechanism of action against the pathogenic causes of each of these conditions. Mass azithromycin treatments did not change the distribution of causes of death among deceased children, perhaps because most deaths were due to infectious causes and were therefore potentially susceptible to the broad-spectrum activity of azithromycin. If mass azithromycin distribution results in a general reduction in child deaths due to infectious causes, then this antibiotic would be expected to be most effective in settings with a high burden of mortality due to infectious diseases, such as in Niger.
