# Supplementary material for: Cause-specific mortality of children younger than 5 years in communities receiving biannual mass azithromycin treatment in Niger: verbal autopsy results from a cluster-randomised controlled trial
Source: Lancet Glob Health. 2020 Jan 22;8(2):e288–95. doi: 10.1016/S2214-109X(19)30540-6 (PMC7025321; doi:10.1016/S2214-109X(19)30540-6)
Supplement: Supplementary appendix [file mmc1.pdf]

THE LANCET
Global Health

Supplementary appendix

This appendix formed part of the original submission and has been peer reviewed. We post it as supplied by the authors.

Supplement to: Keenan JD, Arzika AM, Maliki R, et al. Cause-specific mortality of children younger than 5 years in communities receiving biannual mass azithromycin treatment in Niger: verbal autopsy results from a cluster-randomised controlled trial. *Lancet Glob Health* 2020; **8:** e288–95.

ONLINE MATERIAL

Table of Contents

List of Investigators………………………………………………………………………... 2
eFigure 1…………………………………………………………………………………………..................................... 3
eTable 1…………………………………………………………………………………………..6
eTable 2…………………………………………………………………………………………..7

MORDOR-Niger Study Group Investigators

University of California, San Francisco, San Francisco, CA, USA
Catherine Cook, Sun Y Cotter, Thuy Doan, Dionna M Fry, Jeremy D Keenan, Elodie Lebas, Thomas M Lietman, Ying Lin, Kieran S O'Brien, Catherine E Oldenburg, Travis C Porco, Kathryn J Ray, Philip J Rosenthal, George W Rutherford, Benjamin Vanderschelden, Nicole E Varnado, Lina Zhong, Zhaoxia Zhou

The Carter Center, Atlanta, GA, USA
E Kelly Callahan, Aisha E Stewart

The Carter Center Niger, Niamey, Niger
Ahmed M Arzika, Sanoussi Elh Adamou, Nana Fatima Galo, Fatima Ibrahim, Salissou Kane, Mariama Kiemago, Ramatou Maliki

Programme National de Santé Oculaire, Niamey, Niger
Amza Abdou, Boubacar Kadri, Nassirou Beido

London School of Hygiene and Tropical Medicine, London, UK
Robin L Bailey, John Hart

Johns Hopkins University, Baltimore, MD, USA
Jerusha Weaver, Sheila K West

International Trachoma Initiative, Decatur, GA, USA
Paul M Emerson

Steering Committee. The steering committee for the trial consisted of Robin L Bailey, Jeremy D Keenan, Thomas M Lietman, Travis C Porco, and Sheila K West.

Sponsor program officers. The program officers from the trial's sponsor included Rasa Izadnegahdar, Julie Jacobson, Thomas Kanyok, and Erin Shutes (Bill & Melinda Gates Foundation, Seattle, WA, USA).

Data and Safety Monitoring Committee (DSMC). The trial's DSMC consisted of Judd L Walson (University of Washington, Seattle, WA, USA), Allen W Hightower (Centers for Disease Control and Prevention, Atlanta, GA, USA), Emily E Anderson (Loyola University, Chicago, IL, USA), Wondu Alemayehu (Fred Hollows Foundation, Addis Ababa, Ethiopia), and Latha Rajan (Tulane University, New Orleans, LA, USA).

eFigure 1. Definitions of causes of death, as assessed from the World Health Organization 2007 verbal autopsy instrument. The grey numbers below each box refer to the questionnaire number from the verbal autopsy instrument. Causes of death are listed in order of the hierarchy.

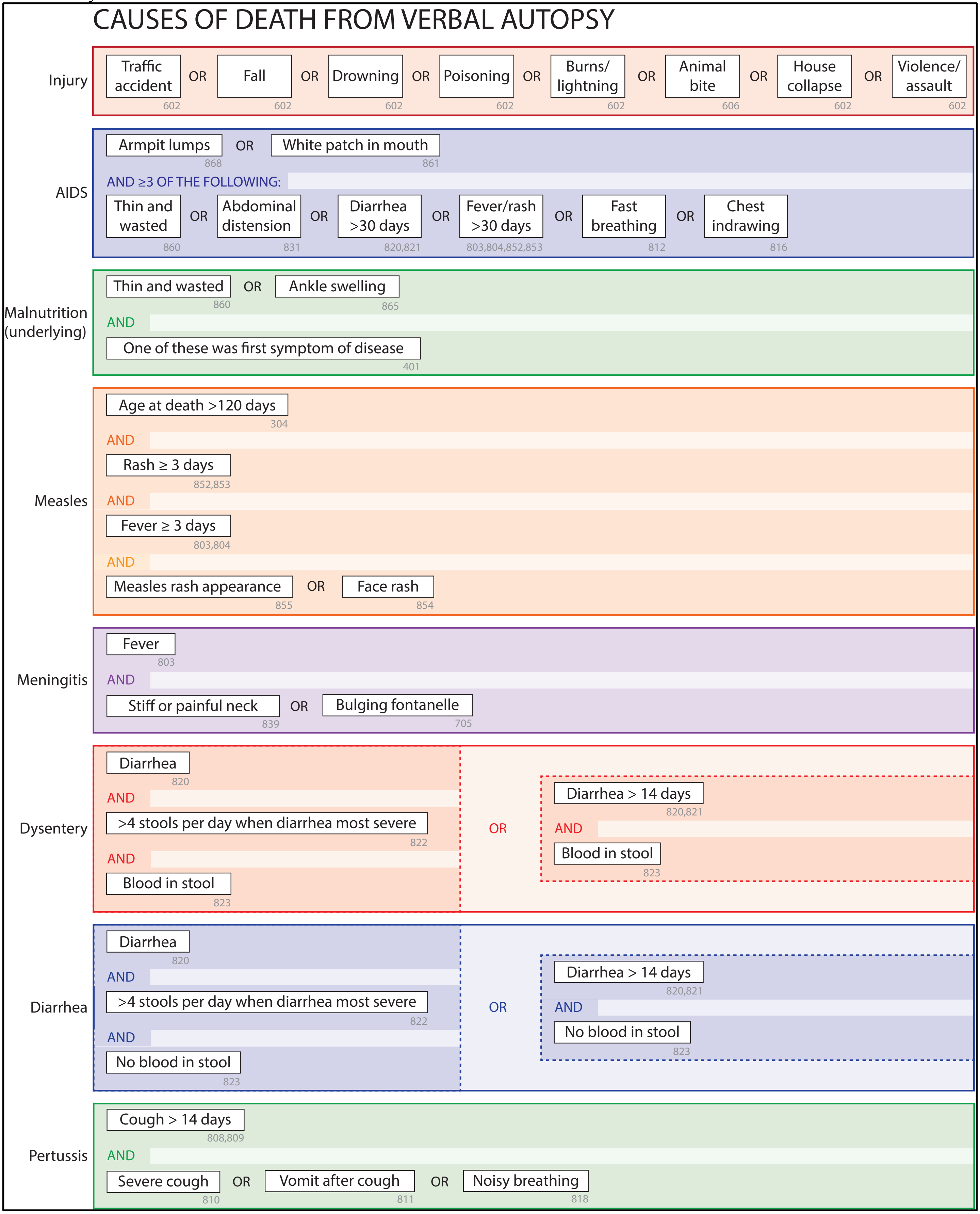

eFigure 1, continued.

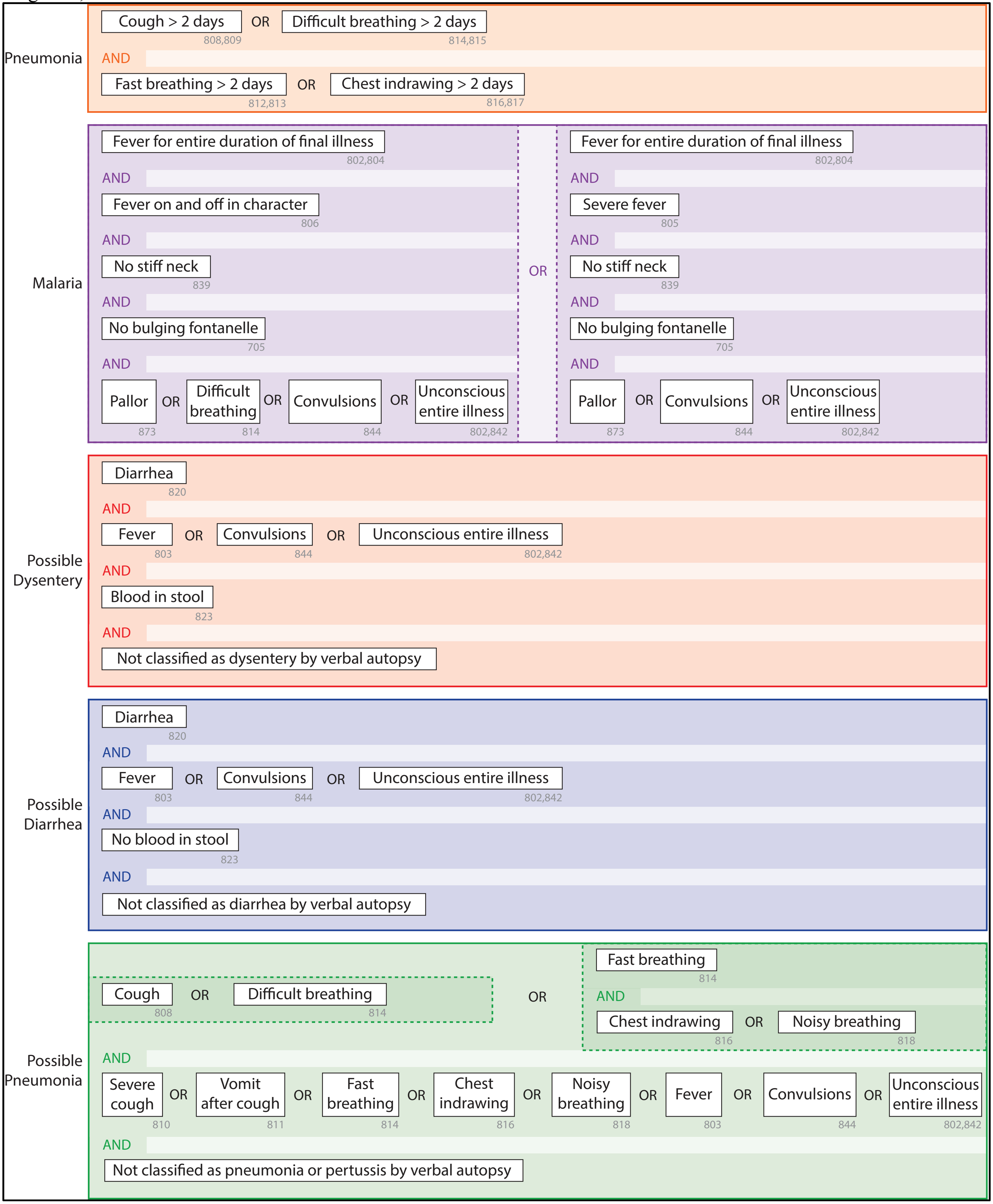

eFigure 1, continued.

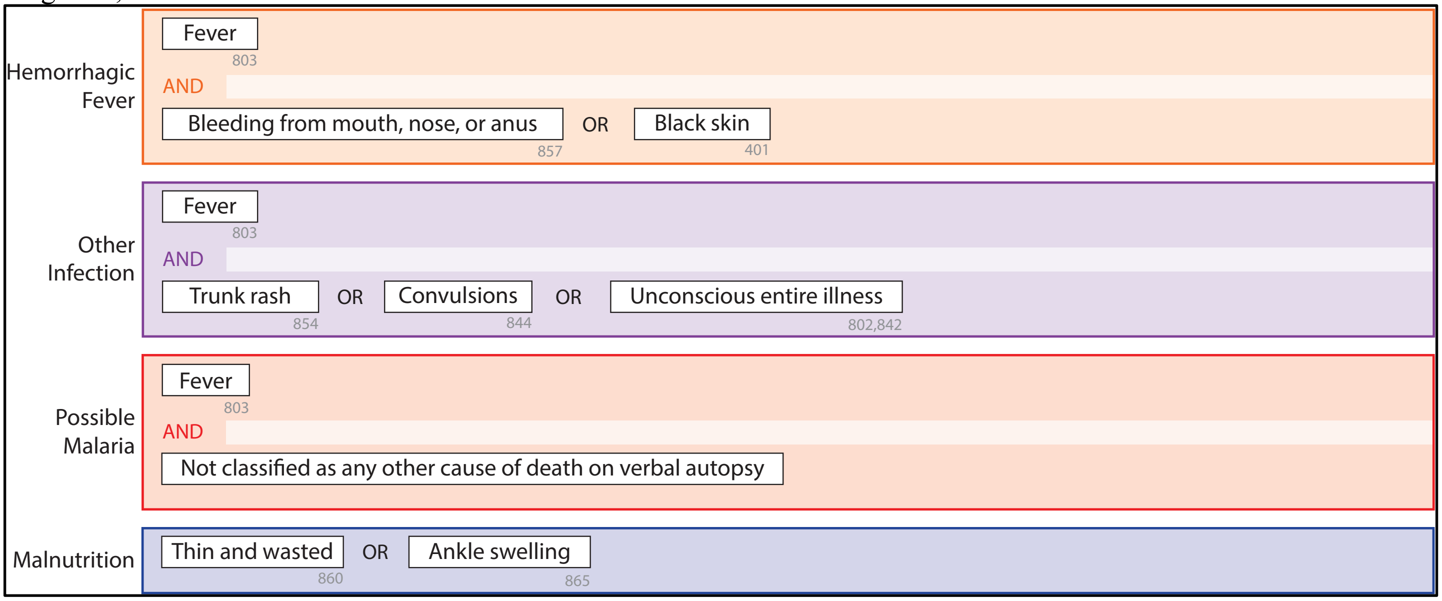

eTable 1. Items included in analysis of raw verbal autopsy data

No.	Question	Response
302	Was the deceased female or male?	Female / Male
502	Did s/he suffer from heart disease?	Yes / No
503	Did s/he suffer from diabetes?	Yes / No
504	Did s/he suffer from asthma?	Yes / No
505	Did s/he suffer from epilepsy?	Yes / No
506	Did s/he suffer from malnutrition?	Yes / No
507	Did s/he suffer from cancer?	Yes / No
509	Did s/he suffer from tuberculosis?	Yes / No
510	Did s/he suffer from HIV/AIDS?	Yes / No
511	Did s/he suffer from any other medically diagnosed illness?	Yes / No
601	Did s/he suffer from any injury or accident that led to her/his death?	Yes / No
801	How is the mother's health now?	Healthy / Ill or dead
803	Did s/he have a fever?	Yes / No
808	Did s/he have a cough?	Yes / No
812	Did s/he have fast breathing?	Yes / No
814,818,819	Did s/he have difficulty breathing, noisy breathing (grunting or wheezing), or flaring of the nostrils?	Yes / No
820	Did s/he have diarrhea?	Yes / No
824	Did s/he vomit?	Yes / No
827	Did s/he have abdominal pain?	Yes / No
830	Did s/he have abdominal distension?	Yes / No
834	Did s/he have any mass in the abdomen?	Yes / No
836	Did s/he have headache?	Yes / No
839	Did s/he have a stiff or painful neck?	Yes / No
841	Did s/he become unconscious?	Yes / No
844	Did s/he have convulsions?	Yes / No
846	Did s/he have paralysis of the lower limbs?	Yes / No
849	Was there any change in the amount of urine s/he passed daily?	Yes / No
852	During the illness that led to death, did s/he have any skin rash?	Yes / No
856	Did s/he have red eyes?	Yes / No
857	Did s/he have bleeding from the nose, mouth, or anus?	Yes / No
858	Did s/he have weight loss?	Yes / No
861	Did s/he have mouth sores or white patches in the mouth or on the tongue?	Yes / No
863	Did s/he have any swelling?	Yes / No
866	Did s/he have any lumps?	Yes / No
869	Did s/he have yellow discoloration of the eyes?	Yes / No
871	Did her/his hair color change to reddish or yellowish?	Yes / No
873	Did s/he look pale (thinning/lack of blood) or have pale palms, eyes, or nail beds?	Yes / No
875	Did s/he have sunken eyes?	Yes / No
901	Was s/he vaccinated for measles?	Yes / No
902	Did s/he receive any treatment for the illness that led to death?	Yes / No
909	Did s/he have any operation for the illness?	Yes / No

eTable 2. Number of children receiving study drug in each treatment arm

Study drug status	Mean (standard deviation)	
	Azithromycin N=303 communities	Placebo N=291 communities
Received study drug		
Month 0 census	130 (91)	119 (85)
Month 6 census	123 (86)	115 (81)
Month 12 census	129 (92)	119 (82)
Month 18 census	121 (85)	111 (77)
Did not receive study drug		
Month 0 census	3 (11)	3 (14)
Month 6 census	12 (12)	10 (12)
Month 12 census	5 (7)	5 (8)
Month 18 census	10 (11)	9 (11)
